# *Anopheles culicifacies* breeding in polluted water bodies in Trincomalee District of Sri Lanka

**DOI:** 10.1186/1475-2875-12-285

**Published:** 2013-08-19

**Authors:** Nayana Gunathilaka, Thilan Fernando, Menaka Hapugoda, Rajitha Wickremasinghe, Panduka Wijeyerathne, Wimaladharma Abeyewickreme

**Affiliations:** 1 Molecular Medicine Unit, Faculty of Medicine, University of Kelaniya, Ragama 11010, Sri Lanka; 2 Department of Parasitology, Faculty of Medicine, University of Kelaniya, PO Box 6, Road, Ragama 11010, Sri Lanka; 3 Department of Public Health, Faculty of Medicine, University of Kelaniya, Ragama 11010, Sri Lanka; 4 Tropical Environmental Diseases & Health Associates, No 3 Elibank Rd, Colombo 5, Sri Lanka

**Keywords:** *Anopheles*, Malaria, Waste water

## Abstract

**Background:**

*Anopheles culicifacies*, the major vector of malaria in Sri Lanka, is known to breed in clean and clear water. The main objective of the study was to detect the breeding habitat diversity of *An*. *culicifacies*.

**Methods:**

Potential larval habitats for *Anopheles* mosquitoes were surveyed on a monthly basis for 17 months (January 2011–June 2012) in four different selected sampling sites (Murthankulam, Kommnaimottai, Paranamadawachchiya and Kokmotawewa) in Trincomalee District of Sri Lanka.

**Results:**

A total of 2,996 larval specimens representing 13 *Anopheles* species were reported from 16 different breeding habitats. According to density criterion, *An*. *culicifacies*, *Anopheles subpictus*, *Anopheles barbirostris*, *Anopheles peditaeniatus* and *Anopheles nigerrimus* were dominant. *Anopheles nigerrimus*, *An*. *subpictus* and *An*. *peditaeniatus* were observed as constant in relation to their distribution. The most productive breeding site for *An*. *culicifacies* was drains filled with waste water in remote areas; the second highest productivity was found in built wells.

**Conclusions:**

These results indicate that *An*. *culicifacies* has adapted to breed in a wide range of water bodies including waste water collections although they were earlier considered to breed only in clean and clear water.

## Background

Sri Lanka embarked on a malaria elimination programme in 2009 with the objective of preventing indigenous transmission of the disease that has been recorded for centuries during more than 100 years of organized modern control efforts. Malaria was endemic mostly in the dry zone of the country and was a leading cause of morbidity and mortality during the past [1]. Malaria transmission depends on a number of hydrology-driven factors that affects the vector survival, including the presence of suitable habitats for the development of anopheline larvae. In urban centres, polluted water is believed to be a major factor that hinders development of anopheline larvae of most of the anopheline vectors. There is evidence that these *Anopheles* mosquitoes’ breeding sites decrease from rural to urban areas [2].

*Anopheles culicifacies*, the major Sri Lankan malaria vector, is known to breed in temporary clean and clear water [3]. As these conditions are seen most in rural areas, the Malaria Control Programme focuses on rural areas in the dry zone for entomological studies. As a result, the bio-ecology of *Anopheles* breeding habitats in urban areas has received very little attention.

Understanding types of larval habitat are important in designing malaria control programmes [4]. Hence, the aim of this study was to describe the productivity of waste water collections as anopheline larval breeding habitats in the Trincomalee District of Sri Lanka.

## Methods

Trincomalee District is situated in the dry zone of the country within the Eastern Province of Sri Lanka, of 2,727 km^2^ and 135/km^2^ of population density is situated in Dry Zone of Sri Lanka, attaining 24.8°C – 30.7°C average temperature and 1, 649 mm annual rainfall. The district has been traditionally endemic for malaria. However, very few entomological investigations have been carried out for about three decades in the Northern and Eastern Provinces (including Trincomalee District), until 2009, due to the civil war that took place in the country.

**Figure 1:**
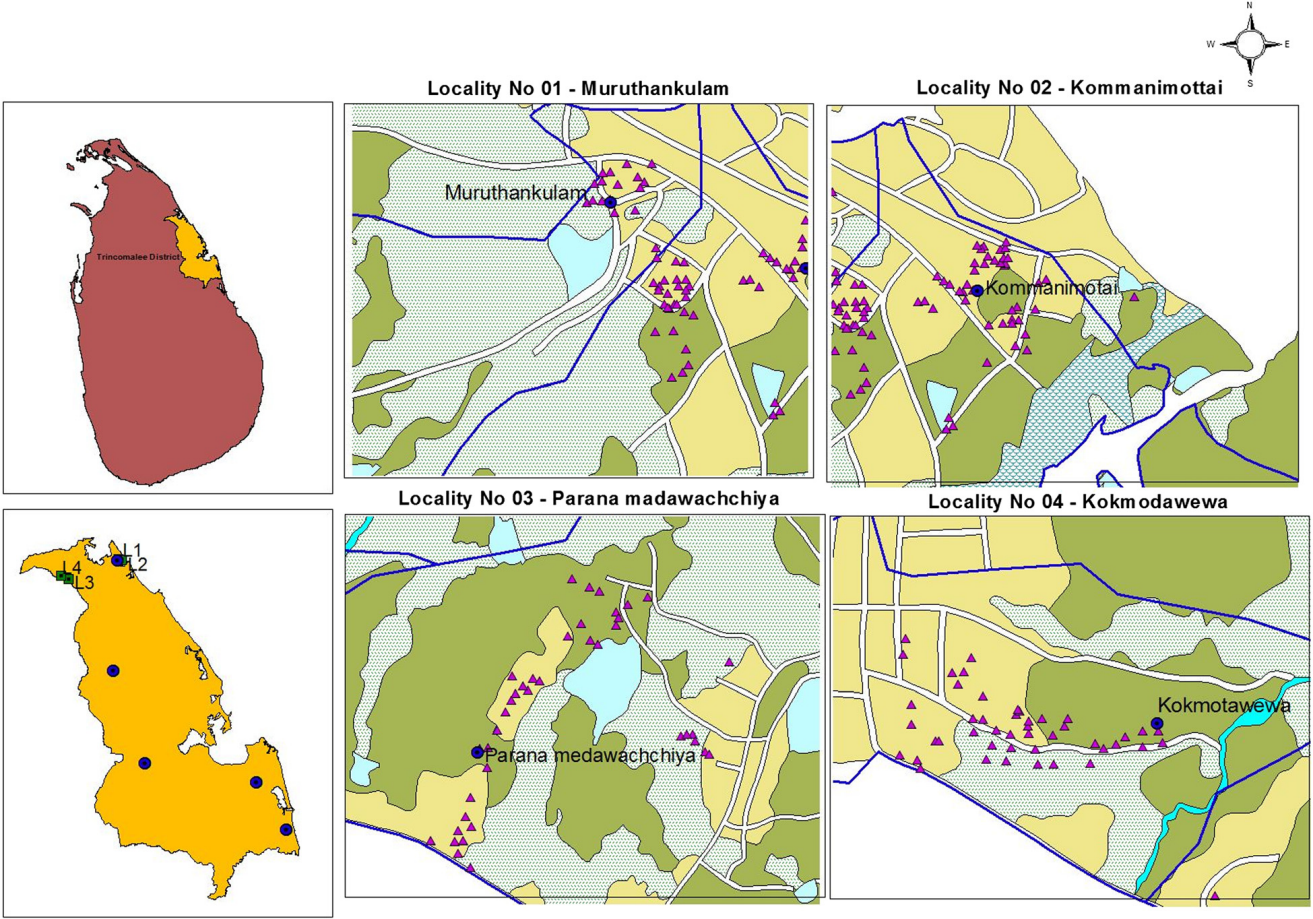
Area map of Padavisiripura sentinel site in Trincomalee District.

Potential mosquito breeding habitats were identified at selected localities through a survey conducted from August-December 2010. The localities were situated within a 20 km radius of sentinel sites located in Gomarankadawala, Ichchallampaththu, Mollipothana, Thoppur and Padavisiripura. Of the five sentinel sites, Padavisiripura sentinel site was observed for conducive breeding of anopheline in a variety of breeding habitats. A cross-sectional survey was carried out between January 2011 and July 2012 in Murthankulam, Kommanimottai, Paranamadawachchiya and Kokmotawewa localities (Figure 1).

### Mosquito sampling

Collection of immature mosquitoes was made by dipping methods as per WHO guidelines [1]. The *Anopheles* larvae were separated from the Culicine larvae. The *Anopheles* mosquito larvae were classified as early instar stage (I and II) or late instar stage (III and IV). The *Anopheles* larvae age grading was done according to Gillies and Coetzee [5].

### Sample identification

Collected stage III and IV larvae were placed individually in a depression microscopic slide with a minimum amount of water and identified under a light microscope with an objective (x l0). I and II instar larvae were reared to reach III and IV instar larvae which were then identified using standard key developed for Sri Lankan *Anopheles*[6].

### Collection of water samples

Three water samples were collected from the breeding habitats of built wells and drains concurrently with the collection of mosquito immature, between 09:00 – 12:00 hr on each sampling day.

### Analysis of water quality parameters

Six abiotic variables; temperature, hydrogen ion concentration (pH), conductivity, salinity, total dissolved solids (TDS), turbidity and dissolved oxygen (DO) were measured on–site at the time of collection. Hydrogen ion concentration was measures using the Hach SenSION TM portable meter, while conductivity, TDS and salinity were measured using the Hach SenSION TM multi probe meter. Turbidity of the water samples were detected using the Hach 2100Q digital meter.

### Data analysis

Seasonal dynamics of mosquito larvae populations in the sampling sites were analysed using the following factors:

*Distribution* was determined as the percent of sampling sites in which a species was noted, according to the formula:

Where:

$$C=\frac{n}{N}\cdot 100\%$$

C - Distribution, n - number of sites of the species, N - Number of all sites.

The following distribution classes were adopted [5].

C1 - sporadic appearance (constancy 0–20%)

C2 - infrequent (20.1 - 40%)

C3 - moderate (40.1 - 60%)

C4 - frequent (60.1 - 80%)

C5 - constant (80.1 - 100%)

*Density* was expressed as percent of specimens of the species in the whole sample according to the formula [7].

$$D=\frac{1}{L}\cdot 100\%$$

Where:

D- Density, l- Number of specimens of each mosquito species, L- Number of all specimens.

The following density classes were accepted.

satellite species (D < 1%)

subdominant species (1 < D <5%)

dominant species (D > 5%)

## Results

Mosquito larval habitat ecology is important in determining larval densities and species assemblage. This in turn influences malaria transmission in an area. Understanding larval habitat ecology is therefore important in designing malaria control programmes. Describing larval habitat characteristics in terms of environmental attributes and identifying relationships between biotic and abiotic factors are important for developing novel methods of vector control in communities with a high propensity to harbour *Anopheles* mosquitoes.

The abundance of *Anopheles* mosquitoes has not been studied in some parts of Sri Lanka especially in the Northern and Eastern Provinces in a systematic manner over the past 30 years due to terrorist activity in the area. Changing weather patterns and ecological changes due to many factors, including climate change, may have resulted in mosquito species shifting their ecological niches to reduce competition for wide dissemination. The present study was conducted to determine larval habitat preferences, densities and diversity of anopheline mosquitoes.

*Anopheles culicifacies* is the primary vector of malaria in Sri Lanka and is known to breed primarily in association with stream and river systems. It prefers clear, sunlit, fresh waters. Intense breeding of *An*. *culicifacies* occurs in sand and rock pools of drying rivers and the margins of slow-moving waterways such as streams and irrigation channels [8-10].

A total of 2,996 larval specimens representing 13 *Anopheles* species were reported from 16 types of breeding habitats. Relative abundance of anophelines encountered are given in Table 1; 44.0% (n = 1,319) larvae were *An*. *culicifacies*, and 19.2% (n = 547) were *Anopheles subpictus*.

**Table 1:** Relative abundance of anopheline larvae at selected localities in the Trincomalee District of Sri Lanka

**Species**	**No of mosquitoes**	**Percentage (%)**
*An*. *culicifacies*	1319	44.03
*An*. *subpictus*	574	19.16
*An*. *annularis*	16	0.53
*An*. *vagus*	48	1.60
*An*. *varuna*	12	0.40
*An*. *tessellatus*	5	0.17
*An*. *nigerrimus*	261	8.71
*An*. *barbirostris*	394	13.15
*An*. *barbumbrosus*	9	0.30
*An*. *jamesii*	5	0.17
*An*. *pallidus*	38	1.27
*An*. *peditaeniatus*	308	10.28
*An*. *pseudojamesi*	7	0.23

Based on the density criterion, *An*. *culicifacies* (44.0%), *An*. *subpictus* (19.2%), *Anopheles barbirostris* (13.2%), *Anopheles peditaeniatus* (10.28%) and *Anopheles nigerrimus* (8.7%) were classified as in the dominant class (D > 5%): *Anopheles vagus*, *Anopheles pallidus* were classified as in the subdominant class (1 < D < 5%); *Anopheles annularis*, *Anopheles varuna*, *Anopheles barbumbrosus*, *Anopheles pseudojamesi*, *Anopheles jamesii* and *Anopheles tessellatus* were classified as satellite species (D < 1%)(Table 2). *Anopheles nigerrimus*, *An*. *subpictus* and *An*. *peditaeniatus* were considered as constant (C = 80.1-100%). Only *An*. *vagus* was considered a frequent (C = 60.1–80%) species. All other anopheline, including *An*. *culicifacies* were considered as infrequent species (C = 20.1–40%). *Anopheles jamesii*, *Anopheles tessellatus*, *Anopheles annularis* and *Anopheles pseudojamesi* were observed as sporadic (Table 3).

**Table 2:** Larval densities of each mosquito species collected from different breeding habitat categories

**Species**	**Waste water**	**Built wells**	**Earth wells**	**Agricultural wells**	**Rainwater collection**	**Animal hoof prints**	**Burrow pits**	**Rock pool**	**Canal**	**Irrigation canal**	**Lake margin**	**Tank margin**	**Pond margin**	**Marshy land**	**Paddy field**	**Slow-moving water**	**Total (%)**
*An*. *culicifacies*	81.6	63.5	4.7	0.0	6.7	0.0	2.0	0.0	0.0	0.0	0.0	0.4	0.0	0.0	0.0	0.0	44.0
*An*. *subpictus*	15.0	11.5	42.3	0.0	34.8	70.6	35.9	0.0	26.7	7.4	7.4	24.3	13.3	46.2	26.7	26.2	19.2
*An*. *annularis*	0.0	0.0	0.0	0.0	0.0	0.0	0.0	0.0	0.0	3.7	0.0	3.3	0.0	0.0	0.0	0.0	0.5
*An*. *vagus*	0.0	0.7	3.4	0.0	0.0	17.6	3.5	10.0	13.3	0.0	3.7	2.2	33.3	0.0	13.3	2.4	1.6
*An*. *varuna*	0.0	0.1	0.0	22.2	1.1	0.0	0.4	0.0	0.0	0.0	7.4	1.1	0.0	0.0	0.0	0.0	0.4
*An*. *tessellatus*	0.0	0.1	0.7	0.0	0.0	0.0	0.0	0.0	6.7	0.0	0.0	0.4	0.0	0.0	0.0	0.0	0.2
*An*. *nigerrimus*	0.5	4.2	12.8	77.8	5.6	11.8	21.5	30.0	46.7	14.8	63.0	15.2	6.7	46.2	46.7	11.9	8.7
*An*. *barbirostris*	2.0	11.6	33.6	0.0	0.0	0.0	22.7	0.0	0.0	0.0	0.0	24.6	0.0	0.0	0.0	50.0	13.2
*An*. *barbumbrosus*	0.0	0.3	0.0	0.0	0.0	0.0	0.8	0.0	0.0	0.0	0.0	0.2	0.0	0.0	13.3	0.0	0.3
*An*. *jamesii*	0.0	0.0	0.0	0.0	0.0	0.0	0.0	0.0	0.0	0.0	3.7	0.7	6.7	0.0	0.0	0.0	0.2
*An*. *pallidus*	0.0	0.2	0.0	0.0	2.2	0.0	0.8	0.0	0.0	22.2	0.0	5.4	6.7	0.0	0.0	0.0	1.3
*An*. *peditaeniatus*	0.9	7.7	2.7	0.0	49.4	0.0	12.5	60.0	6.7	51.9	14.8	20.5	33.3	7.7	0.0	9.5	10.3
*An*. *pseudojamesi*	0.0	0.0	0.0	0.0	0.0	0.0	0.0	0.0	0.0	0.0	0.0	1.6	0.0	0.0	0.0	0.0	0.2
**Total** (%)	100	100	100	100	100	100	100	100	100	100	100	100	100	100	100	100	100

**Table 3:** **Distribution of *Anopheles *species**

**Species**	**Distribution (%)**	**Distribution class**
*An*. *culicifacies*	37.5	C2
*An*. *subpictus*	81.25	C5
*An*. *annularis*	12.5	C1
*An*. *vagus*	68.75	C4
*An*. *varuna*	37.5	C2
*An*. *tessellatus*	18.75	C1
*An*. *nigerrimus*	100	C5
*An*. *barbirostris*	37.5	C2
*An*. *barbumbrosus*	25	C2
*An*. *jamesii*	18.75	C1
*An*. *pallidus*	37.5	C2
*An*. *peditaeniatus*	81.25	C5
*An*. *pseudojamesi*	6.25	C1

Sixteen aquatic habitats were observed; these included waste water, built wells, earth wells, agricultural wells, rain water collections, animal footprints, burrow pits, rock pools, canals, irrigation canals, lake margins, tank margins, pond margins, marshy lands, paddy fields, and slow-moving water streams (Table 2).

Cement bounded wells which use only for drinking, bathing or domestic purposes were considered as built wells. Earth wells were regarded as unbounded wells which use only for drinking, bathing or domestic purposes. Bounded or unbounded wells, which use only for agricultural purposes, were taken as agricultural wells.

In this study, all 13 anopheline species reported were breeding in tank margins; *An*. *nigerrimus* was the predominant species in all habitats. *Anopheles nigerrimus*, *An*. *peditaeniatus*, *An*. *subpictus* and *An*. *vagus* were collected from the same habitats.

There was no habitat found to have only a single species of mosquitoes. Some species, such as *An*. *annularis*, *An*. *varuna*, *An*. *tessellatus*, *An*. *barbirostris*, *An*. *barbumbrosus*, *An*. *pallidus* and *An*. *pseudojamesi* were limited to selective breeding habitats. Different species may have their own habitat range depending on the quality and condition of the water preferred by each species, such as dissolved oxygen (DO), nutrient level, pH, temperature, etc. [11].

There was a habitat partitioning in the study area, implying that mosquito species share food resources within the same habitats. The present study also reports more diversity in *Anopheles* larval compositions as compared to previous studies [12].

In this study, aquatic habitats were varied, which probably made larval abundances significantly different. Breeding sites, such as tank margins and built wells, were more conducive for *Anopheles* larval breeding. However, agricultural wells, rock pools, marshy lands and animal footprints were less productive for larval breeding. *Anopheles culicifacies* was found breeding in waste water, built wells, rainwater pools, earth wells, burrow pits and tank margins. Waste water collections were found from semi-urbanized areas in Murthankulam and Kommanimottai localities, which contained stagnant water in blocked drains contaminated with kitchen and bathing waste. Most of these concrete drains were not covered; some of the drains with concrete slab covers, with open spaces as small as 15 cm between the covers, were positive for *An*. *culicifacies* breeding. These breeding habitats were contaminated with organic litter. *Anopheles subpictus* (14.96%), *An*. *barbirostris* (2.05%), *An*. *peditaeniatus* (0.94%), *An*. *nigerrimus* and *Culex tritaeniorhynchus* were co-breeding with *An*. *culicifacies* in these habitats.

Previous studies conducted in Sri Lanka evidence that *An*. *culicifacies* occurred in habitats with a higher DO and the presence of *An*. *culicifacies* is positively associated with DO [3]. However, the presence study demonstrates that the occurrence of *An*. *culicifacies* can be seen in drains with low DO levels (3.28 ± 0.02 mg/l) and high turbidity (117.1 ± 60.2 NTU). The turbidity of some breeding habitats was detected above the level of 180 NTU. Conductivity of the water in the drains were ranged from 4770 – 1557 μs/cm, which were in the rage of second and third class water quality standards for surface water [13]. In addition, some breeding sites showed third class water quality levels with relevant the level of TDS (>2000 ppm). Therefore, the findings reported here are the first, from Sri Lanka, that demonstrate the ability of *An*. *culicifacies* to breed in drains with low DO, high turbidity and conductivity (Table 4).

**Table 4:** **Mean values of physico**-**chemical parameters in water samples collected from built wells and drains**

**Parameter (Unit)**	**Breeding habitat**	
	**Built wells (n** **=** **25)**	**Drains (n** **=** **20)**
Turbidity (NTU)	9.050 ± 0.636	117.1 ± 60.2
Conductivity (μs/cm)	2475 ± 403	2686 ± 950
Salinity (mg/l)	1256 ± 226	1363 ± 505
Total Dissolved Solids (TDS) (mg/l)	2475 ± 403	1803 ± 740
pH	8.0 ± 0.84	8.46 ± 0.41
Total Dissolved Oxygen (DO) (mg/l)	14.24 ± 0.56	3.282 ± 0.582

Mean ± standard deviation.

*Anopheles culicifacies* were also detected breeding in built wells (63.5%) most of which are used for drinking and bathing purposes. These breeding habitats had higher DO levels with compared to the water in drains (14.24 ± 0.56 ppm). There were no *An*. *culicifacies* larvae found from abandoned built wells and agricultural wells. There is molecular evidence to show that *An*. *culicifacies* sibling species B and E are present in Sri Lanka [14]. *Anopheles culicifacies* sibling E considered the principal vector for *Plasmodium vivax* and *Plasmodium falciparum* breeds in wells [14]. Hence, *An*. *culicifacies* found in wells may be of sibling species E. However, the species breeding in waste water may be sibling species B or another species from Sri Lanka.

The possible explanation as to why *An*. *culicifacies* larvae were frequently found in domestic water containing drains and wells may be due firstly to *An*. *culicifacies* preferentially selecting small, open habitats for oviposition. Secondly, larval predation may be less prevalent in temporary and manmade habitats than it is in large, permanent habitats.

The adaptation and survival of *An*. *culicifacies* and *An*. *subpictus* in polluted water across these areas should be considered a potential facilitator to the emergence of urban malaria in Sri Lanka, a phenomenon that has not been reported on a regular basis as yet. This factor needs to be cautiously taken into consideration by vector control authorities, bearing in mind that the majority of malaria cases reported recently in the country are imported cases detected in people visiting urban areas. Moreover, adaptation of anophelines to breed in polluted water in urban areas could be a serious concern when *Anopheles stephensi* plays an important role in transmitting malaria in neighbouring southern parts of India.

Therefore, the use of BTI or larvicides is recommended for the larval control in these breeding habitats in a cost effective manner. Anyhow, it is warranted to device an appropriate vector controlling measure by the Ministry of Health.

## Conclusions

The present study reveals, for the first time in Sri Lanka, the ability of *An*. *culicifacies* and other potential malaria vectors to breed in drains containing waste water. This requires entomological surveillance in urban areas to detect potential transmission of urban malaria which has not been reported in Sri Lanka.

## Competing interests

The authors declare that they have no competing interests.

## Authors’ contributions

NG - sample collection, identification of field samples and writing the manuscript; TF - sample collection and recording of data; MH - identification of field samples; RW - identification of field samples and data analysis; PW - funding for the research activity and reviewing the manuscript; WA - supervision of the surveillance programme and reviewing the manuscript. All authors read and approved the final manuscript.
